# Maxillary anterior en masse retraction using different antero-posterior position of mini screw: a 3D finite element study

**DOI:** 10.1186/s40510-016-0143-z

**Published:** 2016-10-03

**Authors:** Zohreh Hedayati, Mehrdad Shomali

**Affiliations:** Orthodontic Research Center, School of Dentistry, Shiraz University of Medical Sciences, Shiraz, Iran

## Abstract

**Background:**

Nowadays, mini screws are used in orthodontic tooth movement to obtain maximum or absolute anchorage. They have gained popularity among orthodontists for en masse retraction of anterior teeth after first premolar extraction in maximum anchorage cases. The purpose of this study was to determine the type of anterior tooth movement during the time when force was applied from different mini screw placements to the anterior power arm with various heights.

**Methods:**

A finite element method was used for modeling maxillary teeth and bone structure. Brackets, wire, and hooks were also designed for modeling. Two appropriate positions for mini screw in the mesial and distal of the second premolar were designed as fixed nodes. Forces were applied from the mini screw to four different levels of anterior hook height: 0, 3, 6, and 9 mm. Initial tooth movement in eight different conditions was analyzed and calculated with ANSYS software.

**Results:**

Rotation of anterior dentition was decreased with a longer anterior power arm and the mesial placement of the mini screw. Bodily movements occurred with the 9-mm height of the power arm in both mini screw positions. Intrusion or extrusion of the anterior teeth segment depended on the level of the mini screw and the edge of the power arm on the *Z* axis.

**Conclusions:**

According to the findings of this study, the best control in the sagittal plane during anterior en masse retraction was achieved by mesial placement of the mini screw and the 9-mm height of the anterior power arm. Where control in the vertical plane was concerned, distal placement of the mini screw with the 6-mm power arm height had minimum adverse effect on anterior dentition.

## Background

Three-dimensional control of teeth during orthodontic treatment is important to avoid any side effects on the dentition which might happen due to the applied orthodontic force. In many patients with class II malocclusion or dento-alveolar protrusion, the treatment plan often includes the extraction of the bilateral maxillary first premolars, followed by retraction of the anterior teeth with maximum anchorage (en masse retraction) [1]. In such circumstances, the major orthodontic treatment goal is to reduce the proclination of the maxillary incisors, and therefore, stability of anchorage is crucial in the success of treatment.

Obtaining maximum or absolute anchorage has always been a difficult goal for orthodontists to reach, often resulting in a condition called anchorage loss. Anchorage loss is the reciprocal reaction of the anchor unit which restricts the success of orthodontic treatment by complicating antero-posterior correction [2].

In order to reinforce anchorage, numerous conventional methods such as adding more possible teeth to the anchor unit, increase of torque, the use of trans-palatal arch, Nance holding appliance, different types of headgear, J hooks, cortical anchorage, and inter-arch elastics have been attempted. However, there are inherent problems with these methods consisting of clinical time waste, the need for patient cooperation, and precise wire bending [3–6].

Conventional en masse retraction produces extrusion of the upper anterior teeth, and thus, the application to patients with vertical growth or deep overbite or gummy smile may cause unfavorable results [7].

In order to overcome the problems of conventional anchorage, in recent years, titanium screws have gained enormous popularity in orthodontics and are being considered as absolute sources of orthodontic anchorage [8–10]. These screws have many clinical applications, such as canine retraction, en masse retraction of all anterior teeth together, intrusion of anterior and posterior teeth, and distalization of molars. They are also crucial for allowing the movement of the target tooth with no adverse side effects on other teeth.

As mini screws do not require the patient’s cooperation, they are convenient and time saving resulting in appropriate movement. These screws have always offered sufficient anchorage stability while allowing easy removal without fracturing after treatment [11, 12].

Both inter-radicular spaces between premolar-molar and premolar-premolar were shown to be safe and having enough bone for mini screw insertion [13, 14].

Control of movement of anterior teeth is an essential item for the clinician to obtain an ideal result. The use of power arms enables the orthodontist to achieve controlled movement of the anterior teeth [15]. Force applied from the mini screw can displace and rotate the anterior teeth during retraction in the sagittal and vertical planes. Changing the height of the anterior hook (power arm) can also alter the whole biomechanics paradigm and greatly affect the pattern of teeth movement.

Finding the relation between force direction and tooth displacement allows us to choose appropriate the mini screw position and power arm height for favorable and successful tooth movement.

The finite element method (FEM), which was introduced to calculate initial tooth movement immediately after applying force, has become a useful technique for simulating the pattern of orthodontic tooth displacement [16].

Due to the limitation of clinical trials [17], some studies were conducted by changing the height of the mini screw [16, 18] and the anterior power arm [15] using FEM analysis.

This investigation aims to identify the type of anterior segment movement during en masse retraction using different antero-posterior positions of the mini screw in combination with different vertical heights of the anterior hook.

## Methods

The geometric models of the maxillary dental arch except for the first premolars were constructed [19]. These teeth were arranged in an ovoid arch form. The designed tip and torque of the teeth are shown in Table 1 [20].

**Table 1:** Tip and torque of the teeth

Tooth	Torque	Tip
Central	14′	5′
Lateral	7′	8′
Canine	−3′	10′
Second premolar	−7′	0′
First molar	−10′	10′

**Fig. 1:**
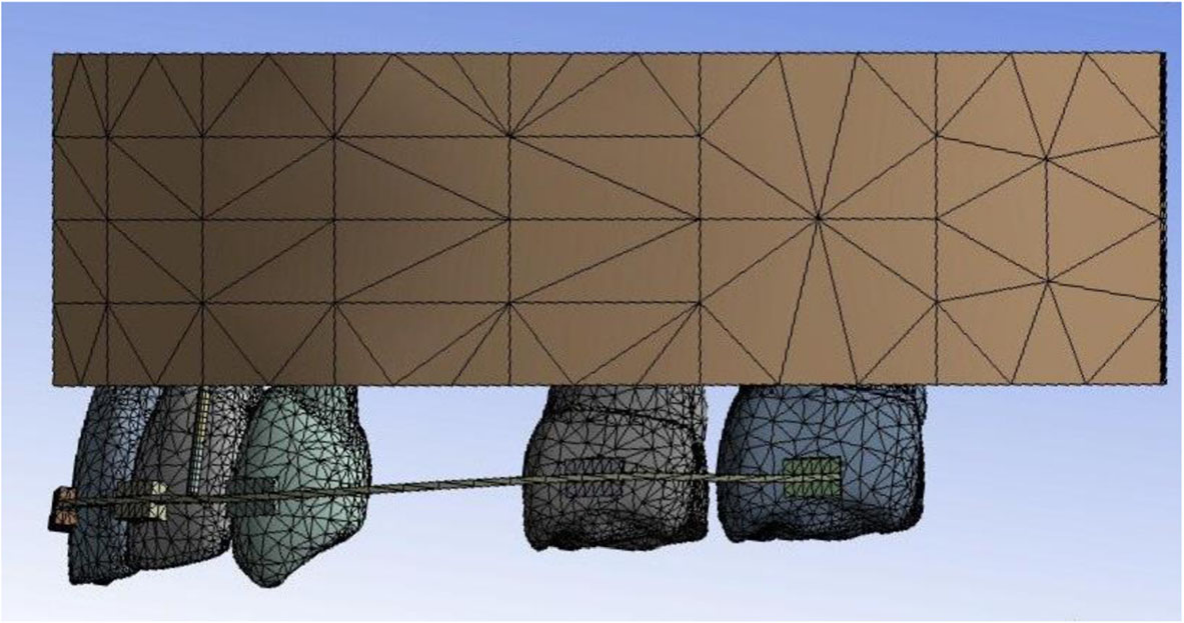
Geometric models of the maxillary dental arch

**Fig. 2:**
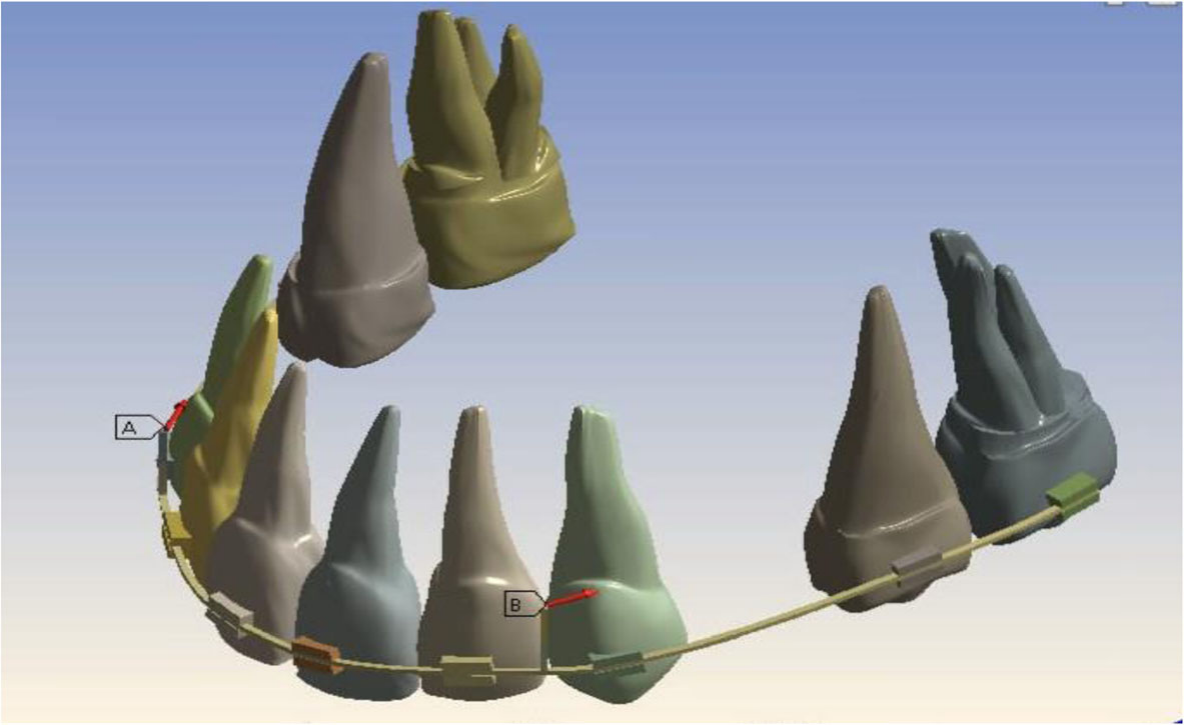
Geometric models of the teeth, wire, bracket, and hook

In order to establish the natural anatomy, periodontal ligaments (PDL) were constructed as a linear elastic film with an average thickness of 0.25 mm around the roots of all the teeth. In the next step, alveolar bone was constructed. Then PDL and the teeth were fitted into the bone (Figs. 1 and 2). Young’s modulus of alveolar bone was greater than PDL and elastic deformation of alveolar bone was insignificant; therefore, the alveolar bone was designed as a rigid body when stress in PDL was calculated.

In order to produce sliding mechanics, brackets with slot size of 0.018 (3M Unitek) were designed and attached to the buccal surfaces of the teeth (4 mm from incisal edge of the central incisor and premolar, 3.5 mm from the incisal edge of the lateral incisor and molar).

A 0.016 in. × 0.022 in. (3M Unitek) stainless steel arch wire with anterior hook (made from stainless steel (0.016 in. × 0.022 in.)) was bonded to the arch wire between the lateral incisor and canine; these combinations were placed on the brackets. The properties of the materials used in this study are summarized in Table 2.

**Table 2:** Material properties used in FEM model

Material	Young’s modulus (Mpa)	Poisson’s ratio
Tooth	2 × 10^4^	0.30
PDL	0.68	0.49
Alveolar bone	2 × 10^3^	0.30
Bracket	2.1 × 10^5^	0.30
Arch wire/hook	2.1 × 10^5^	0.30

The mini screw was placed 6 mm above the arch wire in two different positions: mesial of the second premolar (mesial) and between the second premolar and first molar (distal). Because of their stability in bone, fixed nodes were used as the mini screws [21, 22].

The calculations of the amount and direction of orthodontic tooth movement are based on the resorption and apposition of the alveolar bone (bone remodeling). The bone remodeling rate is assumed to be in proportion to the mean stress in the periodontal ligaments [23].

Anterior en masse retraction was accomplished with 150 gr/side force vectors [24, 25] from the mini screw in two buccal locations to four different levels of anterior hook height: 0, 3, 6, and 9 mm.

Friction between the bracket slots and arch wire was considered during initial tooth movement (μs = 0.74) [26]. The FEM analysis was carried out using ANSYS software (version 12.0.1). The approximate number of nodes is shown in Table 3.

**Table 3:** Approximate number of nodes

Structure	Node number
Bone	151,000
PDL	6500
Teeth	200,000
Hook	500 to 1600
Bracket	12,000
Wire	1100
Total	371,100

## Results

Tables 4 and 5 show the initial tooth movement on the *Y* (sagittal) and *Z* (vertical) axes in two different positions of the mini screw. All numbers were expressed in meters.

**Table 4:** Crown and root tipping in the mesial position of the mini screw

Movement	Tooth	Axis	Height of anterior power arm			
			0 mm	3 mm	6 mm	9 mm
Crown	Central	*Z*	1.02E−06	6.14E−07	1.16E−08	−2.23E−07
	Lateral	*Z*	1.09E−06	7.32E−07	3.07E−08	−2.67E−07
	Canine	*Z*	8.74E−07	5.27E−07	8.10E−09	−1.69E−07
	Central	*Y*	1.05E−06	7.17E−07	5.03E−07	2.12E−07
	Lateral	*Y*	1.23E−06	7.46E−07	5.58E−07	2.53E−07
	Canine	*Y*	9.08E−07	6.37E−07	4.28E−07	1.49E−07
Apical	Central	*Y*	−6.79E−07	−5.32E−07	−2.31E−07	1.02E−07
	Lateral	*Y*	−7.49E−07	−5.97E−07	−3.03E−07	1.02E−07
	Canine	*Y*	−6.60E−07	−4.22E−07	−1.26E−07	8.03E−08

**Table 5:** Crown and root tipping in the distal position of the mini screw

Movement	Tooth	Axis	Height of anterior power arm			
			0 mm	3 mm	6 mm	9 mm
Crown	Central	*Z*	9.89E−07	5.79E−07	3.57E−08	−2.05E−07
	Lateral	*Z*	1.06E−06	7.06E−07	6.19E−08	−2.32E−07
	Canine	*Z*	8.50E−07	5.05E−07	2.90E−08	−1.38E−07
	Central	*Y*	1.06E−06	7.37E−07	5.36E−07	2.28E−07
	Lateral	*Y*	1.27E−06	7.58E−07	5.72E−07	2.91E−07
	Canine	*Y*	9.31E−07	6.51E−07	4.46E−07	1.61E−07
Apical	Central	*Y*	−7.45E−07	−5.43E−07	−2.51E−07	1.12E−07
	Lateral	*Y*	−7.74E−07	−6.19E−07	−3.12E−07	1.40E−07
	Canine	*Y*	−6.69E−07	−4.52E−07	−1.28E−07	8.39E−08

The evaluation of initial tooth movement in both mini screw placements was as follows: in the vertical plane, the intrusion of anterior dentition with the heights of 0, 3, and 6 mm of the power arm was observed. With 9 mm, the upper edge of the power arm was higher than the mini screw. Therefore, anterior dentition was slightly extruded. Maximum and minimum vertical changes occurred in the lateral incisor and canine, respectively.

In the sagittal plane, tipping of the crowns and roots decreased by increasing the height of the power arm. Similarly, maximum and minimum tipping were seen sequentially in the lateral incisor and canine.

## Discussion

En masse retraction after extraction of the first premolar can be conducted using continuous or segmented mechanics. Conventional methods for anterior en masse retraction in sliding mechanics produce extrusion of the upper incisors and clockwise rotation of the occlusal plane, thus causing problems in applying to patients with vertical dento-alveolar excess or gummy smile [27]. Extrusion of molars is not suitable for hyperdivergence patients. Thus, the employment of an appropriate mechanic that controls the extrusion of molars is essential especially in vertical grower patients. However, using the mini screw for anterior segment retraction has minimum (or no) effects on posterior teeth, reducing the adverse side effects of treatment. Upadhyay et al. [21] reported significant improvement in bi-alveolar protrusion patients who were treated with mini screws. Significant reduction in the vertical dimension by intrusion in the maxillary incisors and molars was also obtained.

The center of resistance (CR) for anterior teeth could not be clearly defined because it would change with tooth movement. Melsen et al. [27] indicated the CR of anterior teeth was located 13.5 mm posteriorly and 9 mm superiorly from the center of the arch wire. True translation will occur if the force passes through the CR whereas if the force vector passes below the center of resistance of anterior dentition, uncontrolled tipping of all anterior teeth would be inevitable. Some other investigators [24, 27, 28] estimated the center of resistance of six maxillary anterior teeth to be 13.5 mm apical and 14 mm posterior to the incisal edge of central incisors.

In order to achieve the desired type of tooth movement, altering the height of the anterior retraction hook can make the force application close to the CR. Moreover, different heights of mini implants quantify the torque control from different levels of force vectors [18].

**Fig. 3:**
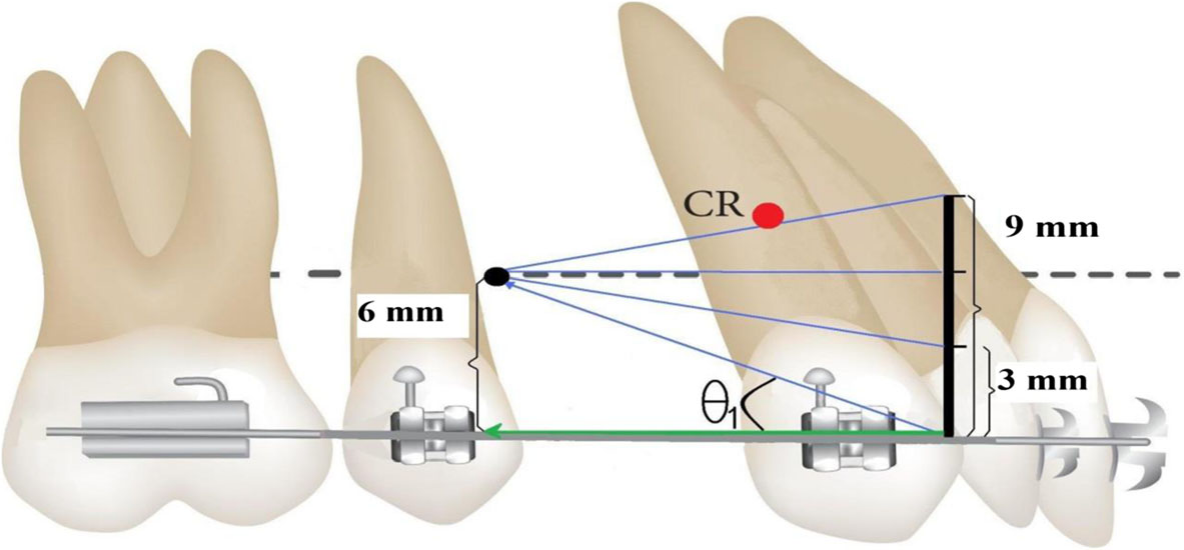
Schematic force diagram and *θ*_1_ angle in the mesial placement of the mini screw

**Fig. 4:**
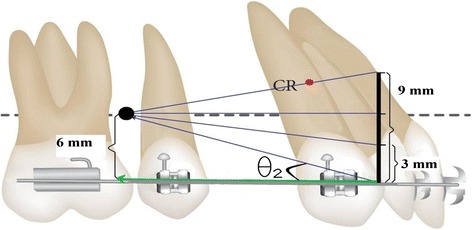
Schematic force diagram and *θ*_2_ angle in the distal placement of the mini screw (*θ*_1_ > *θ*_2_)

Numerous positioning of mini screws have been experimented. Lim [29] stated that in order to improve the vertical force vector, the mini implant should be inserted between the first and second premolars. Lee et al. [30] also reported that greater intrusion of all of the incisor tips and root apexes resulted following insertion of the mini implant into the mesial second premolar area. We employed two appropriate positions for the mini screw in the mesial and distal of the second premolar, 6 mm above the arch wire. Forces were applied from the mini screw to four different levels of anterior hook height: 0, 3, 6, and 9 mm. Force direction from the mini screw to the anterior power arms in this study has been demonstrated in schematic Figs. 3 and 4. In order to conduct en masse retraction of anterior teeth, a force of 150 gm per side was applied and shown to be in physiologic limits for anterior teeth retraction [18, 24, 25].

The angle formed between the force direction of the mini screw and the horizontal component is called the *θ* angle. Changing the *θ* angle will alter the paradigm of biomechanics. Increasing the height of the anterior power arm or the distal placement of the mini screw would cause a decrease in the *θ* angle but would increase the horizontal force (horizontal force = force × cos*θ*). The amount of vertical force is also dependent on the *θ* angle, i.e., decreasing the *θ* angle can reduce the vertical force (vertical force = force × sin*θ*) [16].

In our study, maximum intrusion in both positions of the screw occurred with 0 mm of the power arm (the largest *θ* angle) whereas with 3 mm of the power arm, the intrusion decreased (following decrease in the *θ* angle), and with 9 mm of the power arm, the entire anterior dentition was slightly extruded. Theoretically, according to the *θ* angle, with 6 mm of the power arm, anterior dentition must be neither intruded nor extruded because the position of the mini screw and the edge of the power arm are at the same vertical level (*θ* = 0). But in our finite element analysis, insignificant intrusion was observed which can be interpreted due to some distance from the CR. In addition, when sliding mechanics are employed, the effect of arch wire deflection acting on a tooth can play a role and should be taken into consideration [15].

Mesial displacement (larger *θ* angle) of the mini screw caused greater moment than distal. At a power arm height of 6 mm in combination with distal positioning of the mini screw, minimum effects on the vertical plane resulted. This was consistent with the reports of Lim [29] and Lee et al. [30] which emphasized that insertion of the mini screw between the first and second premolars increases the vertical force vector.

The evaluation of initial tooth movement in the sagittal plane showed that uncontrolled tipping with 0, 3, and 6 mm of the power arm occurred in both positions of the mini screw. Line of action in all these cases passed below the estimated center of resistance of the anterior teeth segment. Obviously, the clockwise moment on the anterior dentition decreases with an increase of the length of the power arm (less distance between the point of action and the center of resistance). These findings are in line with those of the finite element study done by Kojima et al. [23]. They observed that increasing the height of the power arm reduced the clockwise moment of the anterior teeth segment during retraction.

During en masse retraction in the case of the 9-mm power arm, bodily movement (unequal crown and root tipping in the same direction) occurred as the total force passes close to the estimated center of resistance of the anterior teeth [30].

Slight extrusion happened when applying force to the 9-mm power arm. It can be assumed that bodily movement in the anterior dentition occurred, but because of the difference in the vertical level of the mini screw and the power arm, some extrusion was observed. In a FEM study conducted by Tominaga et al. [15] at a level of 5.5 mm of the power arm, no rotation was produced and bodily movement of the anterior segment occurred. Lingual root tipping was observed when the retraction arm was above 5.5 mm.

This side effect in the vertical plane is not suitable in patients with deep overbite or gummy smile. However, the long anterior power arm is uncomfortable and requires good patient cooperation.

Our results indicated that rotation and bodily movements of the anterior dentition were more obvious in the distal placement of the mini screw as compared to the mesial placement.

As a result, with patients who need extraction of the first premolar with different discrepancies in the sagittal and vertical planes, a precision treatment plan with fewer adverse side effects should be chosen with respect to the existing malocclusion.

It is, in fact, the patient’s requirements, such as esthetic, occlusion, function, intensity of discrepancy, and comfort, that guide us to choose the best position of the mini screw and anterior power arm height for having a more satisfactory treatment outcome.

Finite element analysis calculated the initial tooth movement by using an accurate method [31]. These useful information increase our knowledge with regard to the response of tooth displacement to a specific force direction, but this might not be enough for predicting orthodontic tooth movement in clinical practice. Finite element is based on mechanical law [32] without considering the oral cavity condition such as saliva, chewing force, and habit.

Geometric modeling of the bone and PDL is a limitation of finite element study. In this study, the bone was modeled as a solid body and the difference between cancellous and cortical bone was not defined. Also, PDL was modeled as a uniform layer with the same thickness, but even through the root, it is not monotonous.

## Conclusions

Maxillary anterior en masse retraction with the mini screw was evaluated by finite element method. The relation between the force direction and different mini screw positions with varied anterior power arm heights was clarified.

When the mini screw was placed in the distal position (between the second premolar and first molar), the rotation of the anterior teeth segment increased while the movement in the vertical plane decreased.

Increasing the length of the anterior power arm decreased the uncontrolled tipping of the anterior dentition, and with 9 mm of the power arm, bodily movement occurred.

According to patient’s preference and treatment plan, the best position of the mini screw and anterior power arm height must be chosen to reduce the possible adverse side effect and hence improve treatment efficiency.
